# Evaluation of the psychometric properties and minimally important difference of the MD Anderson Symptom Inventory for malignant pleural mesothelioma (MDASI-MPM)

**DOI:** 10.1186/s41687-019-0122-5

**Published:** 2019-06-17

**Authors:** Tito R. Mendoza, Loretta A. Williams, Karen N. Keating, Jonathan Siegel, Cem Elbi, Anna K. Nowak, Raffit Hassan, Brian Cuffel, Charles S. Cleeland

**Affiliations:** 1 Department of Symptom Research, The University of Texas MD Anderson Cancer Center, 1515 Holcombe Boulevard, Unit 1450, Houston, TX 77030 USA; 2 Bayer HealthCare Pharmaceuticals, 100 Bayer Boulevard, Whippany, NJ 07981 USA; 3 Faculty of Health and Medical Sciences, UWA Medical School, The University of Western Australia, 35 Stirling Highway, Perth, WA 6009 Australia; 4 Thoracic and Gastrointestinal Malignancies Branch, NCI/CCR, 10 Center Drive, Bethesda, MD 20892 USA

**Keywords:** Malignant pleural mesothelioma, Psychometric properties, Responsiveness, Patient-reported outcome, Phase 2 trial, MDASI

## Abstract

**Background:**

Symptom assessment requires psychometrically validated questionnaires that are easy to use, relevant to the disease, and quick to administer. The MD Anderson Symptom Inventory for malignant pleural mesothelioma (MDASI-MPM) was adapted from the general (core) MDASI to assess the severity of cancer-related and treatment-related symptoms specific to patients with this condition. The MDASI-MPM includes the 13 core MDASI symptoms, which are experienced by most cancer patients, and 6 MPM-specific items developed via qualitative interviewing, a method favored by the US Food and Drug Administration for instrument item generation and development. Qualitative interviewing that summarizes the item generation and development for the MDASI-MPM is detailed in a separate report. The psychometric study reported here was the next step in developing the validation dossier for the MDASI-MPM.

**Results:**

In this secondary analysis of data from a Phase II trial, 248 patients provided MDASI-MPM data at multiple timepoints during therapy. Over time, fatigue, pain, shortness of breath, feeling of malaise, and muscle weakness were consistently the worst symptoms reported; symptoms interfered most with work and general activity and least with relations with others. Cronbach coefficient alpha values for all MDASI-MPM subscales were at least 0.88 at baseline and 0.91 during treatment, indicating good internal consistency reliability. Intraclass correlations of at least 0.86 for all MDASI-MPM subscales administered a cycle apart (*n* = 82) were indicative of good test-retest reliability. Correlations between MDASI-MPM subscales and LCSS-Meso scores were at least 0.70 (*P* < 0.001 for all comparisons). Patients with good performance status had significantly lower scores than did patients with poor performance status (all *P* < 0.05), supporting evidence for known-group validity and sensitivity. Effect-size differences were 0.69 and higher, indicating medium-to-large effects. The minimally important difference in the MDASI-MPM subscales ranged from 1.0 to 1.5 points on a 0–10 scale.

**Conclusions:**

Symptoms specific to a particular cancer, treatment method, or treatment site can be added to the core MDASI to create a tailored, “fit for purpose” instrument. We found the MDASI-MPM to be a valid, reliable, and responsive (sensitive) instrument for assessing the severity of symptoms of patients with MPM and their interference in patients’ daily functioning.

## Background

Data reflecting the patient experience during an oncologic clinical trial is playing an increasing role in how drug-approval agencies, such as the US Food and Drug Administration (FDA) and the European Medicines Agency, consider the overall clinical risks and benefits of new therapeutic agents. This patient experience is best captured by patient-reported outcome (PRO) questionnaires administered repeatedly over the course of a clinical trial.

Patients with malignant pleural mesothelioma (MPM), an aggressive cancer of the lung pleura, report distressing symptoms, impaired functioning, and treatment intolerance. Understanding the symptoms of MPM requires evidence-based documentation of the symptoms that best characterize the disease and how these symptoms change over the course of treatment. Capturing improvement in disease symptoms is one of the 3 core PRO concepts that the FDA is proposing to focus on for labeling considerations [1].

Only three PRO questionnaires have been validated for use in patients with MPM: the European Organization for Research and Treatment of Cancer Core Quality of Life Questionnaire (EORTC QLQ-C30) [25, 28], the EORTC Quality of Life 13-item Lung Cancer-specific Questionnaire (EORTC QLQ-LC13) [26, 28] and the Lung Cancer Symptom Scale (LCSS) for Mesothelioma (LCSS-Meso) [27]. All three of these instruments measure health-related quality of life (HRQOL). None were originally developed with qualitative input from patients with MPM as required by the FDA, and none measure the symptom burden of MPM and its treatment. Taken together, these deficiencies indicate a significant unmet need.

Building on our group’s extensive experience in developing symptom measures, we used a qualitative approach to adapt an existing multisymptom assessment questionnaire, the MD Anderson Symptom Inventory (MDASI), to include additional symptoms specific to the MPM experience. The resulting provisional version of the MDASI-MPM was based on a conceptual model of MPM-related symptom burden and was found to be content valid and amenable for further psychometric testing [2].

We analyzed a dataset from a large Phase II trial in which the provisional MDASI-MPM was administered longitudinally to patients receiving second-line treatment for MPM. We report here the most prevalent symptoms over time and the degree to which symptoms interfered with patient functioning, and we summarize evidence demonstrating the reliability, validity, and sensitivity of the finalized MDASI-MPM. We also propose metrics for minimally important differences in the MDASI-MPM’s severity and interference subscales.

## Methods

### Study participants

Data used in this secondary analysis were from a randomized (2:1 ratio), open-label, active-controlled, Phase II study of intravenous anetumab ravtansine (BAY 94–9343) or vinorelbine in 248 patients with advanced or metastatic MPM who were overexpressing mesothelin and who had progressed on first-line treatment (platinum in combination with pemetrexed, with or without bevacizumab) (ClinicalTrials.gov Identifier: NCT02610140). Patients were allowed to be randomized into the trial only if they demonstrated mesothelin overexpression at a moderate or stronger level in at least 30% of tumor cells. The dataset does not include the treatment arm and therefore the analyst was blinded to this grouping variable.

Patients completed the provisional MDASI-MPM and the Lung Cancer Symptom Scale (LCSS)-Mesothelioma at times when symptoms were expected to change (to test the MDASI-MPM’s sensitivity) or to be stable (to test the MDASI-MPM’s stability). The MDASI-MPM was completed at baseline, on Days 1 and 15 of each cycle up to 3 cycles, and on Day 1 of Cycles 4, 5, and 6. The LCSS-Meso was administered at baseline and on Day 1 of each cycle (except Cycle 1) for up to 6 cycles. Clinicians rated their patients’ performance status at baseline and on Day 1 of each cycle for up to 6 cycles. Tests for sensitivity were performed between baseline and safety follow-up; tests for stability were conducted between Cycle 2 Day 1 and Cycle 3 Day 1. Patients also completed the provisional MDASI-MPM at a safety follow-up, when most patients were experiencing disease progression, which allowed for additional sensitivity estimates. See Table 1.

**Table 1:** Assessment schedule and the number of respondents at each timepoint

Assessment	MDASI-MPM	LCSS-Meso	ECOG PS
Screening/baseline^a^	239	239	239
Cycle 1 Day 1	0	0	234
Cycle 1 Day 8	164	0	0
Cycle 1 Day 15	153	0	0
Cycle 2 Day 1^b^	210	201	214
Cycle 2 Day 8	157	0	0
Cycle 2 Day 15	156	0	0
Cycle 3 Day 1^b^	167	164	173
Cycle 3 Day 8	119	0	0
Cycle 3 Day 15	108	0	0
Cycle 4 Day 1	135	132	140
Cycle 5 Day 1	112	110	117
Cycle 6 Day 1	90	90	91
Safety follow-up^a^	103	101	117

^a^Tests for sensitivity were performed between baseline and safety follow-up

^b^Tests for stability were conducted between Cycle 2 Day 1 and Cycle 3 Day 1

Abbreviations: *ECOG PS* Eastern Cooperative Oncology Group performance status, *LCSS-Meso* Lung Cancer Symptom Scale-Mesothelioma, *MDASI-MPM* MD Anderson Symptom Inventory for malignant pleural mesothelioma

### Measures

#### The MD Anderson symptom inventory

The core MDASI asks patients to rate the severity of 13 disease-related and treatment-related symptoms during the past 24 h [3]. Each symptom (pain, fatigue, nausea, disturbed sleep, distress, shortness of breath, trouble remembering, lack of appetite, feeling drowsy, dry mouth, feeling sad, vomiting, and numbness or tingling) is rated on an 11-point scale ranging from 0 (not present) to 10 (as bad as you can imagine). The MDASI-MPM includes the 13 core MDASI symptoms and 6 MPM-specific items (feeling of malaise, coughing, muscle weakness, trouble with balance or falling, chest heaviness or tightness, and eye problems) that were developed using qualitative interviewing, a method favored by the FDA for item generation and development [4]. A summary of the item generation and development of the draft MDASI-MPM items on the basis of qualitative interviewing results is reported elsewhere [2]. For the psychometric analyses described in this paper, we included the additional symptoms identified during the qualitative development and evaluated psychometric evidence for their inclusion in the MDASI-MPM symptom severity subscale.

Patients also rated the degree to which their symptoms interfered with various aspects of life during the past 24 h, which is represented by the symptom interference items of the MDASI). Each interference item (general activity, mood, normal work [including both work outside the home and housework], relations with other people, walking ability, and enjoyment of life) is rated on an 11-point scale ranging from 0 (did not interfere) to 10 (interfered completely).

In summary, the MDASI-MPM has 19 items that measure symptom severity and 6 items that measure symptom interference. Correspondingly, the ratings in the MDASI-MPM can be averaged into 2 subscale scores: mean severity (the 13 core symptom items plus the 6 MPM-specific items) and mean interference (the 6 interference items only). A composite symptom score that could serve as a basis for developing a responder analysis or as a potential outcome measure in a pivotal trial can also be calculated but is beyond the scope of this paper.

#### Lung Cancer symptom scale

To evaluate the validity of the MDASI-MPM in comparison with an established instrument, we used the LCSS-Meso, an 8-item questionnaire. The LCSS-Meso is a valid and reliable QOL measure that was designed for patients with non-small cell lung cancer and that has been modified for use in patients with MPM. A recent paper [5] presents cognitive debriefing of the LCSS-Mesothelioma. One of the 8 items in the LCSS-Meso is a global QOL rating (rated using a visual analogue scale on a 0–100 scale). We also calculated an average of all 8 items [6].

#### Performance status

Eastern Cooperative Oncology Group performance status (ECOG PS) was used to represent disease severity [7]. ECOG PS is a physician-rated measure of functional ability, ranging from 0 (fully active; able to carry on all predisease performance without restriction) to 4 (completely disabled; cannot perform self-care; totally confined to bed or chair).

### Statistical analysis

All statistical analyses were conducted using Statistical Package of the Social Sciences software version 21.0 (SPSS, Inc.; Chicago, IL, USA). Correlations, means, standard deviations (SDs), ranges, and 95% confidence intervals (CIs) were computed for all symptoms and subscales. Proportions of patients reporting moderate to severe symptoms were calculated and tabulated. We defined a moderate-to-severe symptom as one rated ≥5 on the MDASI’s 0–10 scale, on the basis of results from previous studies showing that “pain at its worst” is related to greater interference with function when rated ≥5 by cancer patients [8–10] and community samples [11]. Severe ratings are those symptoms rated ≥7. These cutpoints have also been applied to other symptoms [12]. Statistical significance was set using a 2-tailed alpha level of 0.05. To address missing data, analyses using complete data and analyses using all available data at each assessment times were compared, as applicable.

#### Reliability of the MDASI-MPM

##### Internal consistency reliability

Internal consistency reliability refers to the extent to which the items in a scale are measuring the same concept. Cronbach coefficient alphas were computed to estimate the internal consistency reliability of the 2 MDASI-MPM subscales: the severity subscale (13 core plus 6 MPM-specific items) and the interference subscale (6 interference items). The criterion for good internal consistency (reliability) requires a Cronbach alpha value of 0.70 or higher [13].

##### Test-retest reliability

Test-retest reliability is typically examined using assessments taken 1 day apart. However, for this study, we used data from assessments made between Cycle 2 Day 1 and Cycle 3 Day 1 to evaluate test-retest reliability, calculated using intraclass correlations, for the 2 MDASI-MPM subscales. We hypothesized that restricting the analysis to patients who reported relative stability over time based on their response to the LCSS-Meso global QOL item should also report stable symptoms. Hence, change of < 10 points on this QOL item was used as indicative of less-than-meaningful change, on the basis of prior research [14, 15].

#### Validity of the MDASI-MPM

##### Criterion (concurrent) validity

Criterion validity refers to the extent to which an instrument correlates with another instrument that measures a similar, but not the same, concept [13]. To show concurrent validity, we correlated MDASI subscale scores and items with the LCSS-Meso average aggregate scores of all 8 items.

##### Known-group (construct) validity

Construct validation requires demonstrating that the instrument measures the underlying construct it is intended to measure [13]. Various methods of establishing construct validity can be used, such as differentiation between groups (known-group validity), factor analysis, and multitrait–multimethod matrices. For this report, independent-sample *t* tests were used to demonstrate known-group validity, which refers to the extent to which an instrument can distinguish between groups known to be clinically different. Effect sizes were calculated to estimate the magnitude of the differences in the 2 MDASI-MPM subscale scores between those with good (0) versus poor (1 and above) ECOG PS [16, 17].

#### Sensitivity of the MDASI-MPM

Sensitivity (responsiveness) is defined as the ability of an instrument’s subscales or items to detect change in outcomes when such change is expected.

We evaluated whether the MDASI-MPM could detect a worsening of symptoms among patients with deteriorating performance status (a clinical estimate of worsening disease status). Specifically, we examined whether the MDASI-MPM could detect whether symptom severity increased for patients whose ECOG PS deteriorated over time.

Note that we used ECOG PS to demonstrate both known-group validity and sensitivity/responsiveness, based on data from patients who had ECOG PS ratings from both baseline and the safety follow-up. The main difference is that to demonstrate sensitivity/responsiveness, we examined change over time in MDASI-MPM subscales for those patients whose performance status deteriorated over time. Change scores and the associated 95% CI for MDASI-MPM subscales were computed, and effect sizes were calculated, to estimate the magnitude of differences in subscale scores and items [16, 17].

#### Estimation of meaningful change for the MDASI-MPM

##### Anchor-based approach

We used the LCSS-Meso global QOL item as an anchor and as a basis for meaningful change evaluations. MDASI-MPM severity and interference subscale scores between baseline and Cycle 2 Day 1 were calculated. A 10-point change or greater on the global QOL item was used as indicative of a meaningful change, on the basis of prior research [14, 15]. Minimally important differences for improvement and worsening were suggested.

##### Distribution-based approach

Although meaningful change evaluations were primarily derived using relevant patient-based anchor, distribution-based method was also used to complement and support the estimates obtained via anchor-based approach [18]. Our estimates for meaningful change were estimated by tabulating one-half SD, one-third SD, and the standard error of measurement for the MDASI-MPM severity and interference subscales at baseline and at Cycle 2 Day 1 [18].

## Results

### Demographic and clinical characteristics

For the 248 patients in the dataset, age ranged from 42 to 84 years, with the median at 66 years. Women comprised 26% of the sample (*n* = 64). Most (94%) were white and 36% (*n* = 90) were fully active in terms of performance status (ECOG PS = 0).

Of the 248 patients, 239 completed the MDASI-MPM and LCSS-Meso and were graded by clinicians using ECOG PS at baseline. See Table 1 for details on the availability of patients at each assessment time.

### Symptom burden of patients with MPM

**Fig. 1:**
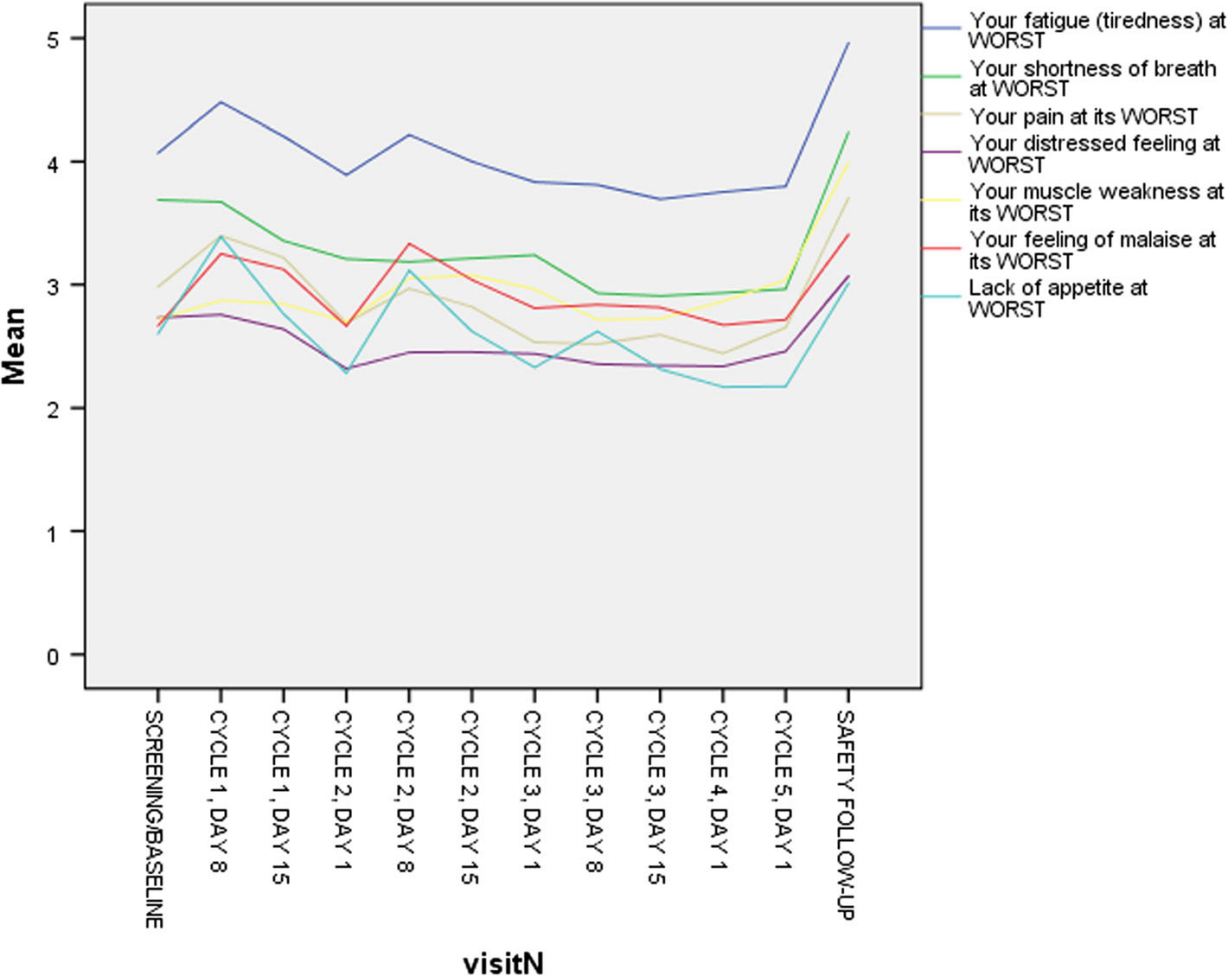
Symptom severity trajectories over time for the top 7 symptoms (using all available data);

Table 2 lists all the MDASI-MPM symptoms, ranked by decreasing severity at baseline and overall across all assessments. At baseline, the most severe symptoms were fatigue, shortness of breath, pain, lack of appetite, feeling of malaise, muscle weakness, and disturbed sleep. Overall, the list and rankings of the worst symptoms are similar. At baseline, 47% of patients had moderate to severe fatigue, with 20% reporting fatigue as severe, and 39% reported having moderate to severe shortness of breath; further, at least 25% of patients also reported having moderate to severe pain, distress, muscle weakness, or feeling of malaise. Overall, 6 symptoms (fatigue, shortness of breath, pain, muscle weakness, feeling of malaise, and disturbed sleep) were moderate to severe for at least 25% of this patient sample. Symptom interference change was observed between baseline and safety follow-up (baseline = 2.8 vs safety follow-up = 4.3, *P* < 0.001; 95% CI, − 2.1 to − 1.0; *n* = 103). Symptoms interfered most with work, followed by general activity, and least with relations with others (Data not shown.). Figure 1 shows the symptom trajectories for the 7 most-severe MDASI-MPM items (fatigue, shortness of breath, pain, distress, muscle weakness, feeling of malaise, and lack of appetite. Over time, fatigue, pain, shortness of breath, feeling of malaise, and muscle weakness were consistently the worst symptoms. Figure 1 also shows that all symptoms were more severe at the safety follow up, which occurred 7 cycles or 21 weeks from baseline, corresponding to disease progression; this difference was significant (baseline = 2.1 vs safety follow-up = 2.9, *P* < 0.001, 95% CI, − 1.2 to − 0.5; *n* = 103).

**Table 2:** Descriptive statistics for MDASI-MPM items at baseline and overall (*N* = 239)

Symptom	Mean	SD	Percent moderate to severe	Percent Severe	Min, max
Baseline					
Fatigue	4.05	2.58	47	20	1, 9
Shortness of breath	3.31	2.63	39	19	1, 10
Pain	2.94	2.69	31	13	1, 10
Distress	2.93	2.57	26	12	1, 10
Muscle weakness	2.89	2.55	26	13	1, 10
Feeling of malaise	2.71	2.59	25	10	1, 10
Lack of appetite	2.66	2.48	24	14	1, 10
Disturbed sleep	2.63	2.70	23	13	1, 10
Feeling drowsy	2.53	2.60	22	13	1, 10
Chest heaviness or tightness	2.24	2.61	19	8	1, 10
Feeling sad	2.20	2.32	22	10	1, 10
Coughing	2.11	2.38	20	7	1, 10
Dry mouth	2.06	2.51	19	9	1, 10
Difficulty remembering	1.88	2.10	16	4	1, 10
Numbness	1.64	2.11	15	7	1, 10
Eye problems	1.43	2.06	8	2	1, 10
Trouble with balance or falling	1.34	2.00	7	4	1, 10
Nausea	1.20	1.90	7	3	1, 9
Vomiting	0.67	1.59	3	2	1, 10
Overall					
Fatigue	4.05	2.58	41	20	1, 9
Shortness of breath	3.31	2.63	31	15	1, 10
Pain	2.94	2.69	26	12	1, 10
Distress	2.93	2.57	22	11	1, 10
Muscle weakness	2.89	2.55	27	14	1, 10
Feeling of malaise	2.71	2.59	26	12	1, 10
Lack of appetite	2.66	2.48	23	12	1, 10
Disturbed sleep	2.63	2.70	25	11	1, 10
Feeling drowsy	2.53	2.60	23	10	1, 10
Chest heaviness or tightness	2.24	2.61	17	7	1, 10
Feeling sad	2.20	2.32	20	10	1, 10
Coughing	2.11	2.38	13	5	1, 10
Dry mouth	2.06	2.51	17	8	1, 10
Difficulty remembering	1.88	2.10	12	5	1, 10
Numbness or tingling	1.64	2.11	16	9	1, 10
Eye problems	1.43	2.06	9	3	1, 10
Trouble with balance or falling	1.34	2.00	10	4	1, 10
Nausea	1.20	1.90	8	3	1, 9
Vomiting	0.67	1.59	4	2	1, 10

Abbreviation: *SD* standard deviation

### Psychometric properties of the MDASI-MPM

#### Reliability

##### Internal consistency reliability

The MDASI-MPM subscales showed good internal consistency reliability. Cronbach coefficient alpha values were at least 0.88 for the severity subscale, and 0.90 for the interference subscale at baseline. Both severity and interference subscale scores were 0.91 or higher during treatment. We found no notable differences in Cronbach coefficient alpha values if only data from the 103 patients who completed the MDASI-MPM at the safety follow-up were used to calculate coefficient alpha values at each assessment time point (Data not shown.).

##### Test-retest reliability

The intraclass correlations of the MDASI severity and interference subscales administered 1 cycle apart for those patients who reported relative stability based on their QOL ratings (*n* = 82) were 0.86 and 0.88, respectively. These values were indicative of good test-retest reliability.

#### Validity

##### Criterion (concurrent) validity

Our analysis of the concurrent validity of MDASI-MPM items showed that the MDASI-MPM subscales were correlated with the LCSS-Meso scores (*P* < 0.001 for all comparisons) (Table 3). For any of the assessments, the correlations between the 2 subscales of the MDASI-MPM and the LCSS-Meso score were higher than 0.70. We found no notable differences in the correlations if only data from the 95 patients who completed both the MDASI-MPM and LCSS-Meso at the safety follow-up were used to calculate correlations at each assessment time point.

**Table 3:** Concurrent validity by correlation of MDASI-MPM subscale scores with LCSS-Meso score at various timepoints

	Sample size	MDASI-MPM symptoms^a^	MDASI-MPM interference^a^
Baseline	238	0.73	0.74
Cycle 2 Day 1	197	0.80	0.75
Cycle 3 Day 1	161	0.78	0.76
Cycle 4 Day 1	131	0.80	0.78
Cycle 5 Day 1	110	0.80	0.77
Safety follow-up	95	0.78	0.79

^a^MDASI-MPM symptoms = the average of the 13 core and 6 MPM-specific items. MDASI-MPM interference = the average of the 6 interference items

Abbreviations: *LCSS-Meso* Lung Cancer Symptom Scale-Mesothelioma, *MDASI-MPM* MD Anderson Symptom Inventory for malignant pleural mesothelioma

##### Known-group (construct) validity

Known-group validity comparisons were made for the MDASI-MPM subscales relative to ECOG PS scores at 6 assessment time points. From baseline to Cycle 3 Day 1, the MDASI-MPM discriminated between patients with good versus poor performance status: patients with good ECOG PS had significantly lower scores for both subscales than did patients with poor ECOG PS (all *P* < 0.05) (Table 4). Similar results were seen for MPM-specific symptoms (all *P* < 0.05). Effect-size differences were 0.65 and higher, indicating medium-to-large effects [16, 17] (Data not shown.).

**Table 4:** Known-group validity by ECOG PS at various timepoints

		MDASI-MPM symptoms^a^			MDASI-MPM interference^a^		
	Sample size	Fully active	Restricted active	95% CI on the difference	Fully active	Restricted active	95% CI on the difference
Baseline	239	1.7 (1.4)	2.5 (1.7)	−1.2 to − 0.4	2.2 (2.1)	3.8 (2.7)	−2.2 to − 0.9
Cycle 2 Day 1	208	1.7 (1.4)	2.3 (1.5)	−1.0 to − 0.1	2.5 (2.3)	3.3 (2.5)	−1.4 to − 0.02
Cycle 3 Day 1	166	1.8 (1.4)	2.3 (1.6)	−1.1 to − 0.06	2.6 (2.2)	3.4 (2.5)	− 1.6 to − 0.03
Cycle 4 Day 1	135	1.9 (1.4)	2.2 (1.6)	−0.8 to 0.24	2.6 (2.3)	3.2 (2.6)	−1.5 to 0.3
Cycle 5 Day 1	112	2.1 (1.5)	2.2 (1.6)	−0.7 to 0.5	2.9 (2.4)	3.3 (2.7)	−1.4 to 0.6
Safety follow-up	95	2.5 (1.1)	2.5 (1.6)	−1.1 to 1.0	3.9 (1.8)	3.6 (1.5)	−1.1 to 1.7

^a^MDASI-MPM symptoms = the average of the 13 core and 6 MPM-specific items. MDASI-MPM interference = the average of the 6 interference items

Abbreviations: *CI* confidence interval, *ECOG PS* Eastern Cooperative Oncology Group performance status, *MDASI-MPM* MD Anderson Symptom Inventory for malignant pleural mesothelioma

#### Sensitivity (responsiveness)

We assessed whether the MDASI-MPM could detect symptom changes when performance status changed during the course of treatment. In the Phase II trial, 95 of the 117 patients with ECOG PS data also had MDASI-MPM data at the safety follow-up; of these, 53% (50/95) showed a decline in performance status.

We found that the increase in the 2 MDASI-MPM subscales were correlated with change in ECOG PS (Table 5). Change scores for patients whose ECOG PS worsened over time were statistically significant for both subscales. With Bonferroni correction for multiple comparisons, MDASI-MPM symptoms, such as trouble with balance or falling, eye problems, muscle weakness, numbness, and dry mouth, were also significantly correlated with change in ECOG PS. These differences were clinically meaningful, as reflected by effect sizes of one-half SD and higher.

**Table 5:** Sensitivity of the MDASI-MPM based on changes in ECOG Performance Status (PS)

	Patients with worsening PS from baseline to safety follow-up (*n* = 50)			
	Baseline Mean (SD)	Safety follow-up Mean (SD)	95% CI	Effect Size
MDASI-MPM subscale				
MDASI-MPM symptoms^a^	2.2 (1.6)	3.2 (1.9)	− 1.5 to − 0.5	0.69
MDASI-MPM interference^a^	2.8 (2.4)	5.0 (2.8)	−3.0 to − 1.4	0.92
MDASI-MPM symptom				
Trouble with balance or falling^b^	0.9 (1.6)	2.7 (3.0)	1.0 to 2.7	1.13
Eye problems^b^	0.5 (1.3)	1.7 (2.2)	0.4 to 1.8	0.92
Muscle weakness^b^	2.4 (2.6)	4.5 (3.3)	1.3 to 2.9	0.81
Numbness^b^	1.7 (2.7)	3.7 (3.3)	1.1 to 2.9	0.74
Dry mouth^b^	1.7 (2.2)	3.0 (2.4)	0.5 to 2.1	0.59
Fatigue	4.2 (2.5)	5.4 (2.5)	0.4 to 2.0	0.48
Feeling sad	1.9 (2.6)	3.1 (3.3)	0.4 to 2.0	0.46
Feeling malaise	2.5 (2.6)	3.7 (3.0)	0.2 to 2.2	0.46
Lack of appetite	2.4 (2.7)	3.5 (2.7)	0.1 to 2.2	0.41
Distress	2.4 (2.4)	3.3 (3.0)	0.1 to 1.6	0.38
Shortness of breath	3.6 (2.4)	4.5 (3.0)	−0.1 to 1.8	0.38
Chest heaviness	2.2 (2.2)	3.0 (2.9)	.01 to 1.5	0.36
Pain	3.5 (2.7)	4.3 (2.9)	−0.1 to 1.6	0.30
Feeling drowsy	2.8 (2.7)	3.5 (2.9)	−0.2 to 1.6	0.26
Coughing	2.7 (2.8)	2.0 (2.2)	−1.4 to 0.1	0.25
Difficulty remembering	1.6 (2.1)	2.1 (2.5)	−0.1 to 1.1	0.24
Disturbed sleep	2.8 (2.9)	3.2 (2.7)	−0.5 to 1.4	0.14
Nausea	1.4 (1.9)	1.2 (1.9)	−0.4 to 0.8	0.11
Vomiting	0.7 (1.5)	0.8 (1.3)	−0.3 to 0.6	0.07

^a^MDASI-MPM symptoms = the average of the 13 core and 6 MPM-specific items. MDASI-MPM interference = the average of the 6 interference items

^b^Significant at *P* < 0.01

Abbreviations: *MDASI-MPM* MD Anderson Symptom Inventory for malignant pleural mesothelioma, *SD* standard deviation

#### Estimation of meaningful change for the MDASI-MPM

Table 6 shows estimates of meaningful change in the MDASI-MPM subscales using the QOL item from the LCSS-Meso as the anchor. Meaningful change estimate for improvement was associated with an approximately 1-point (on a 0–10 scale) improvement in the MDASI-MPM subscales. Table 6 also demonstrates that our estimates for meaningful change via distribution-based methods were approaching 1 point based on tabulated values using one-half SD, one-third SD, and the standard error of measurement for the MDASI-MPM severity and interference subscales at baseline and at Cycle 2 Day 1.

**Table 6:** Meaningful change estimation using 2 different approaches

Anchor-based estimation of meaningful change				
	*n*	Improvement (all *n* = 61)	No change (*n* = 70)	Decline (all *n* = 52)
MDASI-MPM symptoms^a^	183	1.05 (1.96)	0.16 (1.46)	−0.65 (1.30)
MDASI-MPM interference^a^	181	1.07 (3.19)	0.18 (2.19)	−0.92 (2.16)
Distribution-based estimation of meaningful change				
	*n*	One-half SD	One-third SD	SEM
MDASI-MPM symptoms^a^	183	0.79	0.52	0.76
MDASI-MPM interference^a^	181	1.26	0.83	1.27

^a^MDASI-MPM symptoms = the average of the 13 core and 6 MPM-specific items. MDASI-MPM interference = the average of the 6 interference items

Anchor = Global QOL item (0–100 scale) from the LCSS-Meso

Abbreviations: *MDASI-MPM* MD Anderson Symptom Inventory for malignant pleural mesothelioma, *SD* standard deviation, *SEM* standard error of the mean

**Fig. 2:**
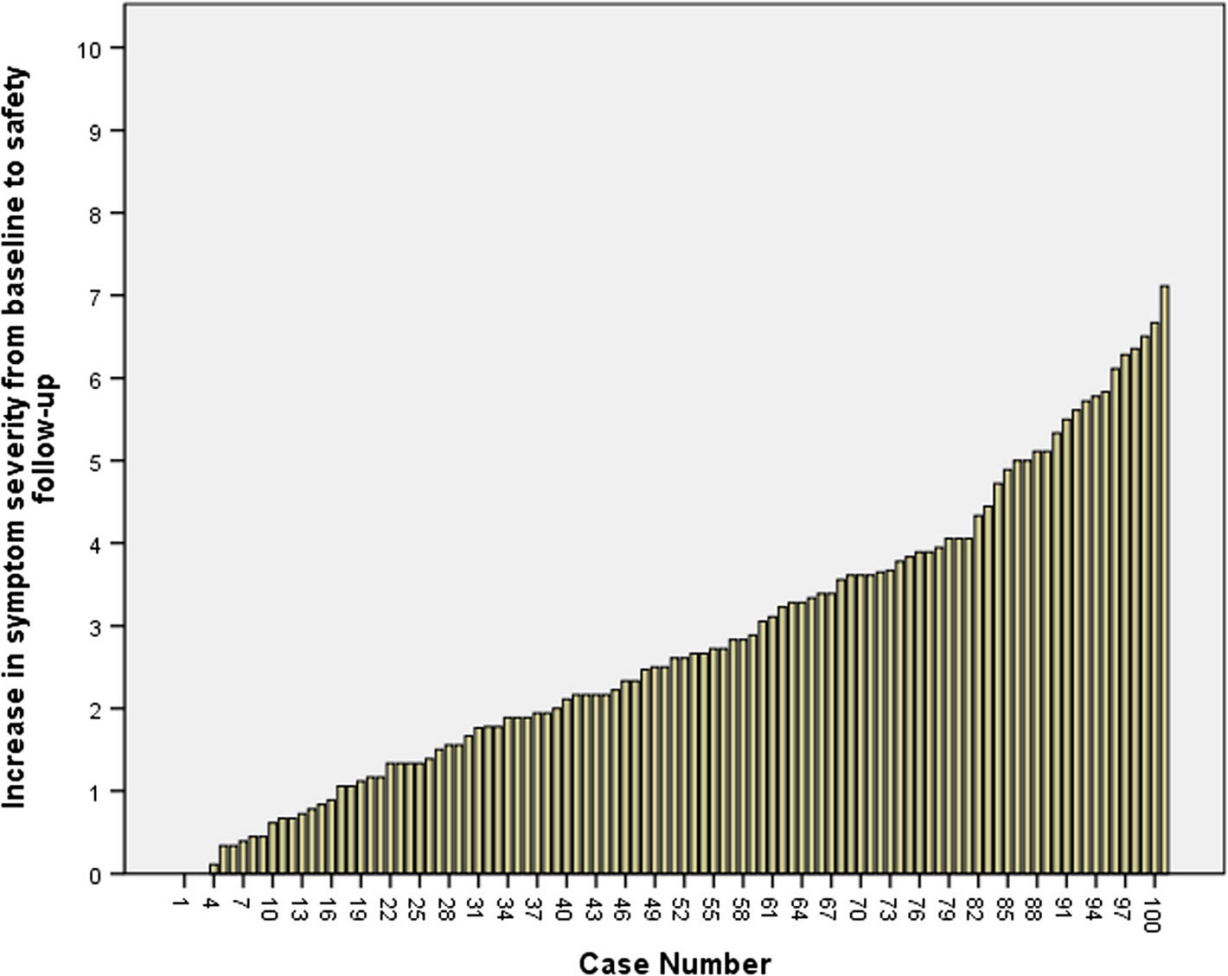
Waterfall plot depicting changes in symptom severity scores from baseline to safety follow-up. All patients who completed both MDASI-MPM assessments at baseline and safety follow-up showed symptom worsening (*N* = 98). About 86% of the patients had a 1-point or greater increase in symptom severity while 65% had a at least a 2-point symptom worsening

Both above approaches are considered group-level because we are examining scores calculated for each relevant group. To illustrate how individual patient’s symptom severity vary from baseline to the safety follow-up, we present a waterfall plot (Fig. 2).

## Discussion

In this study, we tested a provisional MPM-specific version of the MD Anderson Symptom Inventory adapted to encompass major symptoms reported by patients with MPM over the duration of treatment. We adopted all of the MDASI-MPM items for psychometric testing on the basis of qualitative interviewing results that established the importance of each item [2]. This psychometric testing is a necessary step in the validation dossier of the MDASI-MPM. This new MDASI version was administered longitudinally to more than 200 patients in a large Phase II trial, with the analysis being blinded to treatment arm, so as to collect data on its psychometric properties. A limitation of the study is that we were unable to determine if the MDASI-MPM is sensitive to drug-related treatment benefits because we did not have the treatment arm in the analytic dataset.

The results provide psychometric support for the use of the MDASI-MPM in clinical trials in which symptom change is a candidate endpoint. The instrument’s severity and interference subscales exhibited high test-retest reliability and acceptable internal consistency reliability. As expected, QOL ratings from the LCSS-Meso were highly correlated with ratings from the MDASI-MPM severity and interference subscales. The 2 MDASI-MPM subscales, symptom severity and interference with functioning, were sensitive to changes in performance status (related to disease), as evidenced by significant correlations between MDASI-MPM ratings and ECOG PS over time. The 2 MDASI-MPM subscales were also correlated with patient global QOL ratings.

By rank ordering the severity of symptom items across various cancer types, researchers can identify most of the symptoms that are consistently burdensome for patients with cancer. For example, the 7 most severe symptoms reported by our study participants—fatigue, shortness of breath, pain, distress, muscle weakness, feeling of malaise, and lack of appetite—include both core and disease-specific MDASI items that are also used in MDASI modules for other disease sites [19–22]. One advantage to the adaptation of the MDASI for MPM is that having symptom data derived from the core items of the MDASI allows for comparison of common symptoms across disease sites. Another advantage of the MDASI-MPM is its use of a numeric rating scale, which has been shown to offer distinct advantages in measuring symptoms, especially pain [23].

The MDASI-MPM was developed according to the general principles expressed in the FDA’s guidance on the use of PROs in labeling claims [4], including qualitative generation of items. While none of the HRQOL measures validated for use in patients with MPM were developed with qualitative input from patients with MPM, a recent paper [5] evaluated the content validity of the LCSS-Meso in qualitative interviews with patients with MPM, although an expert panel evaluation of the relevance of these symptoms was not done. This study found that the symptoms reported by 20% or more of the 18 patients with MPM interviewed were shortness of breath, fluid build-up, coughing, fatigue, pain, weight loss, and appetite changes. Although fluid build-up was the second most commonly reported symptom (reported by 78% of patients), Gelhorn and colleagues (2018) excluded it along with weight loss as signs observable by clinicians rather than symptoms. The remaining symptoms found by Gelhorn and colleagues (2018) are included in the LCSS-Meso. The qualitative development of the MDASI-MPM found results similar to those of Gelhorn et al. (2018) with the addition of distress, nausea, vomiting, problems with remembering things, and disturbed sleep being reported by 20% or more of patients (Williams et al., 2018). While chest heaviness and tightness, likely the symptom of fluid build-up, was only reported by 10% of the patients, the expert panel who considered the relevance of items for the MDASI-MPM recommended including it. The current study found that the symptom severity of chest heaviness and tightness was similar to coughing (mean = 2.24 and 2.11, % moderate to severe 19 and 20, and % severe 8 and 7 respectively) at baseline, making it an important symptom to assess and monitor. In addition the MDASI-MPM includes symptoms of trouble with balance or falling and muscle weakness, identified by patients in qualitative interviews and recommended by an expert panel for relevance. The current study showed both symptoms to be very sensitive to worsening performance status in patients with MPM. The MDASI-MPM presents a more comprehensive picture of the symptom burden of MPM than the HRQOL measure LCSS-Meso.

The general principles expressed in the FDA’s guidance on the use of PROs in labeling claims [4] also recommends psychometric evaluation of the items and subscales in an early-phase study [2]. One of the criteria set forth in the FDA guidance is that a PRO instrument must be able to detect change over time. In particular, the regulatory agency is interested to see if changes in the scores are related to changes in a patient’s clinical status. We have shown here that the MDASI-MPM is sensitive to changes in performance status (related to disease) and to patients’ QOL ratings.

The MDASI-MPM takes less than 5 min to complete and can be easily adapted for clinical settings. This conciseness makes the MDASI-MPM well suited for frequent administration, which provides rich information about the trajectory of symptoms across the course of treatment. Such longitudinal information can be especially informative for stakeholders such as patients, clinicians, and regulators making decisions about evaluating new cancer therapies [24].

The symptom data from this large Phase II trial also indicate that some symptoms, both core MDASI and MPM-specific items, were relatively infrequently endorsed in the psychometric evaluation. Because these symptoms were also less severe, they could be excluded when developing a composite score as a potential outcome for future clinical trials.

## Conclusion

This study provides psychometric evidence for the use of the MDASI-MPM in tracking changes in MPM symptoms during treatment. The instrument’s severity and interference subscales exhibited high test-retest reliability and acceptable internal consistency reliability. The MDASI-MPM subscales were sensitive to changes in performance status (related to disease), as evidenced by significant correlations between MDASI-MPM ratings and ECOG PS over time and between MDASI-MPM ratings and QOL ratings over time. Our examination of the minimally important differences for the MDASI-MPM subscales should be useful as a guide for those designing clinical trials in which symptom change is a potential endpoint.

## Abbreviations

CI: Confidence interval
ECOG PS: Eastern Cooperative Oncology Group performance status
FDA: US Food and Drug Administration
LCSS-Meso: Lung Cancer Symptom Scale-Mesothelioma
MDASI: MD Anderson Symptom Inventory
MPM: Malignant pleural mesothelioma
PRO: Patient-reported outcome
QOL: Quality of life
SD: Standard deviation
SEM: Standard error of the mean
